# In vitro antibacterial, antioxidant and cytotoxic activity of acetone leaf extracts of nine under-investigated Fabaceae tree species leads to potentially useful extracts in animal health and productivity

**DOI:** 10.1186/1472-6882-14-147

**Published:** 2014-05-05

**Authors:** Jean P Dzoyem, Lyndy J McGaw, Jacobus N Eloff

**Affiliations:** 1 Phytomedicine Programme, Department of Paraclinical Sciences, Faculty of Veterinary Science, University of Pretoria, Private Bag X04, Onderstepoort 0110 Pretoria, South Africa; 2 Present address: Department of Biochemistry, Faculty of Science, University of Dschang, P.O. Box 67, Dschang, Cameroon

**Keywords:** Antioxidant, Antimicrobial, Cytotoxicity, Efficacy, Fabaceae, Safety

## Abstract

**Background:**

The Fabaceae family is the second largest family of medicinal plants, containing more than 490 species which are being used as traditional medicine. The aim of this study was to determine the antioxidant and antibacterial activity as well as the cytotoxicity of acetone leaf extracts of nine tree species from the Fabaceae family that have not been investigated well previously for possible use in animal health and production.

**Methods:**

The antibacterial activity was determined by a serial microdilution method against three Gram-positive and three Gram-negative bacteria. Antioxidant activity was determined using free-radical scavenging assays. The safety of the extracts was ascertained using the 3-(4,5-dimethylthiazol-2-yl)-2,5-diphenyltetrazolium bromide (MTT) assay on Vero African green monkey kidney cells.

**Results:**

Six of the nine acetone extracts had significant antibacterial activity against at least one of the six bacterial species with (MIC 20–80 μg/mL). The *Crotalaria capensis* extract had the highest activity against *Salmonella typhimurium*, followed by *Indigofera cylindrica* with MICs of 20 μg/mL and 40 μg/mL respectively. The *Dalbergia nitidula* extract had free radical scavenging capacity (IC_50_ of 9.31 ± 2.14 μg/mL) close to that of the positive control Trolox in the DPPH assay. The *Xylia torreana* extract also had high activity (IC_50_ of 14.56 ± 3.96 μg/mL) in the ABTS assay. There was a good correlation between antioxidant activity and total phenolic content (R^2^ values > 0.8). The extracts had weak or no toxicity to Vero cells, compared to the positive control doxorubicin with the LC_50_ varying from 10.70 ± 3.47 to 131.98 ± 24.87 μg/mL at the concentrations tested.

**Conclusion:**

Extracts of *D. nitidula, X. torreana, C. capensis* and *I. cylindrica* had a low cytotoxicity and high antimicrobial and/or antioxidant activity. These species are therefore promising candidates for the development of useful antimicrobial/antioxidant preparations with a low cytotoxicity that may be useful in promoting animal health and productivity.

## Background

Most of the potentially harmful effects of oxygen are due to the formation and activity of a number of chemical compounds known as reactive oxygen species. Many such reactive species are free radicals derived either from normal metabolic processes in the human body or from external sources [1]. Free radicals and peroxidative damage have been implicated in several human and animal pathological disorders, including inflammatory ailments and microbial infectious diseases [2]. The deleterious effects of the free radicals can be diminished by natural antioxidants available in plants. Among the various natural compounds extracted from plants that have demonstrated biological activities, many are receiving particular attention as radical scavengers [3]. Therefore, the discovery of novel antimicrobial agents from plants that also target free radicals with a low toxicity may be useful in the field of phytotherapy. Natural products present in higher plants are an important source of therapeutic agents and many research groups are currently screening different biological activities of plants [4]. Several studies have proven the antioxidant or radical scavenging properties of medicinal plant extract [5]. The Phytomedicine Programme of the University of Pretoria (http://www.up.ac.za/phyto) has contributed to this area by determining the antibacterial and antifungal activity of acetone leaf extracts of more than 500 tree species against 8 very important bacterial and fungal pathogens. Nine tree species belonging to the Fabaceae family, with very few or no previous pharmacological investigations reported were selected from the Phytomedicine plant database for in depth investigation.

The Fabaceae is the third largest family among the angiosperms after the Orchidaceae and Asteraceae, consisting of more than 700 genera and about 20 000 species of trees, shrubs, vines, and herbs worldwide [6]. It is the second largest family of medicinal plants, containing over 490 species used as traditional medicine [7]. To the best of our knowledge, the plant species involved in this study are being screened for the first time, except *Erythrina caffra* Thunb., which has been reported to have antimicrobial activity [8]. Some of these species are used traditionally to combat infections (Table 1). In this study, *in vitro* antibacterial and antioxidant activities of the acetone leaf extracts of nine South African medicinal plants belonging to the Fabaceae family were investigated. Because plant material from nearly 290 species belonging to 100 genera of the Fabaceae has been reported to be toxic [7], the cytotoxicity was determined to ascertain the safety of the studied extracts.

**Table 1:** Characteristics of the nine plants of Fabaceae family investigated

**Name of plant**	**Common names**	**Synonyms**	**Parts used**	**Traditional or other uses**	**Voucher number**
*Baphia racemosa* (Hochst) Baker.	Violet pea, Natal camwood (Eng.); boskamhout (Afr.); Tshuphu (isiXhosa); Fithi (Zulu)	*Bracteolaria racemosa* Hochst.	Leaves	Source of food for larvae of the blue-spotted charaxes. Parrots feed on young seeds [9].	PMDN 397
*Crotalaria capensis* Jacq.	Cape rattle-pod, Cape laburnum (Eng.); Kaapse klapperpeul (Afr.); Bukheshezane (Zulu)	-	Flowers	Decorative horticultural value [10].	PMDN 318
*Dalbergia nitidula* Baker	Glossy flat-bean (English) Mudima (Shona) Murima (Shona) Purple-wood dalbergia (English)	*Dalbergia dekindtiana* Harms	Roots	For some species in this genus, a paste of charred and powdered stems mixed with water is used for sore mouths in infants [11].	PMDN 367
*Erythrina caffra* Thunb.	Coast coral tree (Eng.); kuskoraalboom (Afr.); umsinsi (Zulu); umsintsi (Xhosa)	*Erythrina viarium* Tod.	Bark, leaves, roots	Sores, tuberculosis, respiratory infections, wounds, eardrops for earache, sprains [11].	PMDN 280
*Indigofera cylindrica*	Natal Camwood (Eng.) Natal Camwood	*Indigofera frutescens Indigofera jucunda* Schrire	-	Plants of the genus are used for infertility, menstrual cramps, toothbrushes, mouthwash [12].	PMDN 178
*Lonchocarpus nelsii* (Schinz) Heering & Grimme	Apple-leaf lance-pod, Kalahari apple-leaf, Kalahari lance-pod (Eng.)	*Dalbergia nelsii*, *Philenoptera nelsii* (Schinz) Schrire	-	Plants of the genus are used as a laxative, for treating convulsion in infants, and for minor skin trouble [13].	PMDN 612
*Podalyria calyptrata* (Retz.) Willd.	Sweetpea bush, large pink keurtjie, water blossom-pea (Eng.); keur, keurtjie, keurblom, ertjiebos, waterkeurtjie (Afr.)	*Podalyria kunthii* Walp., *Podalyria lanceolata* (E. Mey.) Benth.	-	Purely decorative as no records of other uses could be traced.	PMDN 759
*Virgilia divaricata* Adamson.	Blossom tree (Eng.); keurboom (Afr.)	-	-	Transparent gum exudate from the bark is used as a substitute for starch [14].	PMDN 192
*Xylia torreana* Brenan	Hairy sand ash, Zambezi ash (Eng.)	*Xylia africana* sensu Torre	-	No information on tradition use found	PMDN159

-: not reported, Eng.: English, Afr.: Afrikaans.

Extracts with a good antimicrobial activity or good antioxidant activity and low toxicity may be useful in addressing animal health and productivity in aspects such as microbial infections, replacing antibiotic feed additives and treating infectious diarrhoea, a focus area of the Phytomedicine Programme.

## Methods

### Plant material and extraction

The leaves of plants were collected in the Pretoria National Botanical Garden in South Africa. The identity of the plant material was confirmed by the curator and voucher specimens were placed in the HGWJ Schweickerdt Herbarium of the University of Pretoria (PRU) (Table 1). Collected tree leaves were dried at room temperature in a well-ventilated room and ground to a fine powder in a Macsalab Mill (Model 2000 LAB Eriez). One gram of each plant was extracted in 10 mL of acetone, (technical grade, Merck) in a polyester centrifuge tube. The tube was vigorously shaken for 30 min on an orbital shaker, then centrifuged at 4000 × g for 10 min and the supernatant was filtered using Whatman No.1 filter paper before being transferred into pre-weighed glass containers. This was repeated thrice on the same plant material and the solvent was removed by evaporation under a stream of air in a fume hood at room temperature to produce the dried extract.

### Chemicals

Sodium carbonate was obtained from Holpro Analytic, South Africa. Gentamicin was purchased from Virbac, South Africa. Fetal calf serum (FCS) and minimum essential medium (MEM with L-glutamine) was provided by Highveld Biological, Johannesburg, South Africa. Phosphate buffered saline (PBS) and trypsin were purchased from Whitehead Scientific, South Africa. Doxorubicin was obtained from Pfizer, South Africa. Dimethyl sulfoxide (DMSO), 2,2’-Azino-bis (3-ethylbenzothiazoline-6-sulphonic acid) diamonium salt (ABTS), 2,2-diphenyl-1-picrylhydrazyl (DPPH), 3-4,5-dimethylthiazol-2-yl)-2,5-diphenyltetrazolium bromide (MTT), p-iodonitrotetrazolium violet (INT), Folin-Ciocalteu reagent, gallic acid, 2,5,7,8-tetramethylchroman carboxylic acid (Trolox) and potassium persulfate were purchased from Sigma-Aldrich St. Louis, MO, USA. Müller Hinton agar and broth were from Sigma-Aldrich, India.

### Antibacterial assay

The antimicrobial activity of extracts dissolved in acetone was determined against six bacterial strains: three Gram-positive bacteria (*Staphylococcus aureus* ATCC 29213, *Enterococcus faecalis* ATCC 29212, *Bacillus cereus* ATCC 14579) and three Gram-negative bacteria (*Escherichia coli* ATCC 25922, *Pseudomonas aeruginosa* ATCC 27853 and *Salmonella typhimurium* ATCC 14028). This activity was evaluated by the determining the minimal inhibitory concentration (MIC) using a rapid broth microdilution technique with p-iodonitrotetrazolium violet (INT) as growth indicator as developed by Eloff [15]. The samples were serially diluted to provide a final concentration range of 2.5 to 0.02 mg/mL. In addition to MIC, the total activity was calculated as the total mass in mg extracted from 1 g of plant material divided by the MIC value (mg/mL). The total activity in ml/g indicates the volume to which the extract derived from 1 g of plant material can be diluted and still inhibits the growth of the microorganism [16].

### Antioxidant assays

#### Total phenolic content determination

The total phenolic content was determined colorimetrically using a Folin-Ciocalteu 96-well microplate assay developed by Zhang et al [17]. The total phenolic content was calculated from the linear equation of a standard curve prepared with gallic acid, and expressed as gallic acid equivalent (GAE) per g of extract.

#### ABTS radical assay

The ABTS radical scavenging capacity of the samples was measured with modifications of the 96-well microtitre plate method described by Re et al. [18]. Trolox and ascorbic acid were used as positive controls, methanol as negative control and extract without ABTS as blank. The percentage of ABTS• + inhibition was calculated using the formula: Scavenging capacity (%) = 100 - [(absorbance of sample - absorbance of sample blank) × 100/ (absorbance of control) – (absorbance of control blank)]. The IC_50_ values were calculated from the graph plotted as inhibition percentage against the concentration.

#### DPPH assay

The DPPH radical-scavenging activity was determined using the method proposed by Brand-Williams et al. [19]. Ascorbic acid and Trolox were used as positive controls, methanol as negative control and extract without DPPH as blank. Results were expressed as percentage reduction of the initial DPPH absorption in relation to the control. The concentration of extract that reduced the DPPH colour by 50% (IC_50_) was determined as for ABTS• + .

### Cytotoxic activity

The cytotoxicity of the acetone extracts against Vero monkey kidney cells was assessed by the MTT reduction assay as previously described [20] with slight modifications. Cells were seeded at a density of 1 × 10^5^ cells/ml (100 μl) in 96-well microtitre plates and incubated at 37°C and 5% CO_2_ in a humidified environment. After 24 h incubation, extracts (100 μl) at varying final concentrations were added to the wells containing cells. Doxorubicin was used as a positive reference. A suitable blank control with equivalent concentrations of acetone was also included and the plates were further incubated for 48 h in a CO_2_ incubator. Thereafter, the medium in each well was aspirated from the cells, which were then washed with PBS, and finally fresh medium (200 μl) was added to each well. Then, 30 μl of MTT (5 mg/ml in PBS) was added to each well and the plates were incubated at 37°C for 4 h. The medium was aspirated from the wells and DMSO was added to solubilize the formed formazan crystals. The absorbance was measured on a BioTek Synergy microplate reader at 570 nm. Cell growth inhibition for each extract was expressed in terms of LC_50_ values, defined as the concentration that caused 50% of inhibition of cell viability. The selectivity index (SI) values were calculated by dividing cytotoxicity LC_50_ values by the MIC values (SI = LC_50_/MIC). Tests were carried out in quadruplicate and each experiment was repeated thrice.

### Statistical analysis

All experiments were conducted in triplicate and values expressed as mean ± standard deviation. For the MIC values, re-evaluation of the growth inhibition was conducted where variation within the three experiments was noticed. Statistical analysis was performed using one way ANOVA and results were compared using the Fisher's least significant difference (LSD) at a 5% significance level.

## Results and discussion

### Antimicrobial activity

The MIC values of the leaf extracts of 9 species of the Fabaceae family are presented in Table 2. Extracts had a broad spectrum of activity against all bacterial strains. Crude extracts with an MIC < 100 μg/mL are usually considered as significantly active [21] and therefore could be regarded as promising candidates for further studies. In this study, six of the nine extracts investigated had significant activity, including extracts of *C. capensis* against *S. typhimurium* followed by the extract of *I. cylindrica* with respective MICs of 20 and 40 μg/mL. Significant activities were also recorded (MIC of 80 μg/ml) with extracts of *C. capensis, E. caffra* and *L. nelsii* against *E. faecalis. D. nitidula* and *V. divaricata* had similar activity against *B. cereus.* Olajuyigbe and Afolayan previously reported moderate antibacterial activity of the ethanolic extract from *E. caffra* bark against two Gram positive (*E. faecalis*, *S. aureus*) and two Gram negative (*E. coli, P. aeruginosa*) bacteria (MICs ranged from 156.6 to 625 μg/mL) [8].

**Table 2:** Minimal inhibitory concentration (MIC in μg/mL) and total activity (TA in mL/g) of acetone leaf extracts from nine plants of the Fabaceae family against bacterial strains

**Plant name**	**Extraction yield (%)**	**ME (mg)**	**Microorganisms**											
			***S. aureus***		***E. faecalis***		***B. cereus***		***E. coli***		***P. aeruginosa***		***S. typhimurium***	
			**MIC**	**TA**	**MIC**	**TA**	**MIC**	**TA**	**MIC**	**TA**	**MIC**	**TA**	**MIC**	**TA**
*Baphia racemosa*	3.7	37	310	119.35	160	231.25	625	59.20	625	59.20	>1000	<37	625	59.20
*Crotalaria capensis*	4.6	46	625	73.60	**80**	575	310	148.39	625	73.60	625	73.60	**20**	**2300**
*Dalbergia nitidula*	4.2	42	160	262.5	160	262.5	**80**	525	160	262.5	310	135.48	160	262.5
*Erythrina caffra*	3.9	39	>1000	<39	**80**	487.5	310	125.81	160	243.75	160	243.75	310	125.81
*Indigofera cylindrica*	1.9	19	625	30.40	310	61.29	160	118.75	310	61.29	310	61.29	**40**	475
*Lonchocarpus nelsii*	0.9	9	>1000	<9	**80**	112.5	625	14.40	625	14.40	>1000	<9	**80**	112.5
*Podalyria calyptrata*	3.4	34	>1000	<34	625	54.40	>1000	<34	310	109.68	>1000	<34	160	212.5
*Virgilia divaricata*	8.9	89	310	287.1	625	142.40	**80**	**1112.5**	310	287.1	>1000	<89	**80**	**1112.5**
*Xylia torreana*	1.2	12	310	38.71	160	75	160	75	310	38.71	310	38.71	160	75
Gentamicin	nd	nd	0.2	nd	0.2	nd	0.8	nd	0.4	nd	0.2	nd	1.56	nd

Mass in mg extracted from 1 g (ME).

Values in bold indicate MICs lower than 100 μg/mL and total activity higher than 1000 ml/g, nd: not determined.

A significant antimicrobial activity of an extract is a highly promising result, since an extract consists of a mixture of compounds. A bioassay-guided fractionation might lead to the isolation of active ingredients with an activity better than that of standard antibiotics. However, low activity was noticed with all the extracts against the Gram-positive *S. aureus* and two Gram-negative bacteria, namely *E. coli* and *P. aeruginosa*. The significant activity of extracts against Gram-negative bacteria (including *S. typhimurium*) which are often resistant to antimicrobial agents compared to Gram-positive [22], could be explained by the possibility that the active compounds may act by inhibiting bacterial growth without necessarily penetrating into the bacterial cell itself. Although an interesting activity was found with the extracts of *C. capensis* against *S. typhimurium,* the calculated total activity was lower (475 mL/g) compared to that of the *I. cylindrica* extract (2 300 mL/g). Therefore, the extract of *I. cylindrica* appears to be the most promising antimicrobial candidate for isolation of biologically active compounds as well as for the rational use of the plant extract in primary health care by rural communities for human or animal health.

### Antioxidant activity and total phenolic content

The antioxidant capacity expressed as IC_50_ value and total phenolic content of all extracts are shown in Table 3. All the extracts had moderate to potent antioxidant activity The IC_50_ values occurred in the range of 9.31 ± 2.14 to 271.58 ± 51.96 μg/mL in the DPPH assay, and 14.56 ± 3.96 to 207.09 ± 70.75 μg/mL in the ABTS radical assay. The use of at least two different assays in evaluating antioxidant activity of plant extracts has been recommended by Moon and Shibamoto [23]. Significant variations (p < 0.05) were observed between the extracts tested. With IC_50_ of 9.31 ± 2.14 μg/mL, extracts from *D. nitidula* had free radical scavenging capacity close to that of the positive control Trolox (IC_50_ of 9.71 ± 2.23 μg/mL) in the DPPH assay. A similar result was obtained with the *Xylia torreana* (IC_50_ of 14.56 ± 3.96 μg/mL) extract in the ABTS assay where Trolox had an IC_50_ of 12.48 ± 3.7 μg/mL. These results indicate that these two extracts could be a potential source of natural antioxidants.

**Table 3:** Antioxidant activity and total phenolic content (TPC) of acetone extracts from nine plants of the Fabaceae family

**Plant name**	**DPPH IC**_**50 **_**(μg/mL)**	**ABTS IC**_**50 **_**(μg/mL)**	**TPC (mg GAE/g)**
*Baphia racemosa*	210.69 ± 65.48^a^	195.10 ± 54.68^a^	0.12 ± 0.1^a^
*Crotalaria capensis*	195.26 ± 30.64^a^	207.09 ± 70.75^a^	2.21 ± 0.95^b^
*Erythrina caffra*	268.6 ± 29.69^a^	173.28 ± 43.04^a^	3.29 ± 1.38^c^
*Lonchocarpus nelsii*	247.70 ± 66.47^a^	134.64 ± 16.49^a^	1.51 ± 0.74^b^
*Virgilia divaricata*	271.58 ± 51.96^a^	150.57 ± 19.23^a^	0.86 ± 0.53^d^
*Indigofera cylindrica*	22.31 ± 9.92^b^	41.39 ± 15.74^b^	8.94 ± 1.52^e^
*Xylia torreana*	16.90 ± 5.45^b^	**14.56 ± 3.96**^**c**^	12.05 ± 1.23^f^
*Podalyria calyptrata*	35.21 ± 3.46^c^	36.66 ± 3.59^b^	7.89 ± 1.48^e^
*Dalbergia nitidula*	**9.31 ± 2.14**^**d**^	21.30 ± 5.07^d^	14.39 ± 0.62^g^
Trolox	9.71 ± 2.23^d^	12.48 ± 3.7^c^	nd
Ascorbic acid	2.44 ± 0.9^e^	3.14 ± 1.07^e^	nd

Values in bold indicate MICs lower than 100 μg/mL and total activity higher than 1000 ml/g.

n = 3, means values within a column with different superscript letters are significantly different at p < 0.05; mg GAE/g: mg of gallic acid equivalent per g of dry plant material.

**Figure 1:**
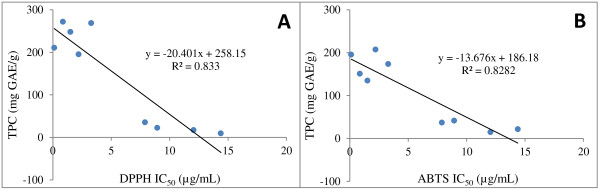
**Correlation between antioxidant capacity and total phenolic content of nine species from the Fabaceae family.** Antioxidant capacity was measured by DPPH assay **(A)** and ABTS radical assay **(B)**. GAE: gallic acid equivalent.

The total phenolic contents (TPC) of the extracts were in the range of 0.12 ± 0.1 to 14.39 ± 0.62 mg GAE/g. According to Makkar [24] the TPC of an extract amounting to 5 mg GAE/g is considered to be significant and could have a beneficial antioxidant efficacy. Taking into account this cut-off, extracts of *D. nitidula*, *I. cylindrica, P. calyptrata* and *X. torreana* contain significant amounts of phenols. The TPC correlated well with the respective antioxidative activity of extracts (Figure 1). Good correlation was found between the TPC and IC_50_ values for both DPPH and ABTS (R^2^ > 0.833 and R^2^ > 0.828 respectively). Polyphenols have been reported to be responsible for the antioxidant activity in plant extracts [25].

The high levels of TPC observed with the extract of *D. nitidula* and *I. cylindrica* could be related to their good antimicrobial activity because many phenolic compounds have antimicrobial activity and antioxidant activity [26]. Although no *in vivo* experiments have been done, the good antioxidant activity of acetone extracts combined with good antibacterial activity indicates that these extracts may fulfill a dual role if used therapeutically.

In the case of *C. capensis, E. caffra, L. nelsii* and *V. divaricata* the good antibacterial activity was apparently not due to the presence of phenolics. Many secondary metabolites other than phenolic compounds are well known for their antimicrobial activities [27].

### Cytotoxic activity

The cytotoxicity was determined using an *in vitro* assay with Vero monkey kidney cells. The LC_50_ and the selectivity index (SI) values were calculated (Table 4). The LC_50_ values ranged from 10.70 ± 3.47 to 131.98 ± 24.87 μg/mL while the SI ranged from 0.003 to 8.25. Extracts of *P. calyptrata* had the highest LC_50_ (lowest toxicity) of 131.98 ± 24.87 μg/mL. *B. racemosa* had an LC_50_ of 10.70 ± 3.47 μg/mL, which is relatively cytotoxic. Our results indicated that all the plant extracts investigated were less toxic to Vero cells than the positive control, doxorubicin. Apart from *B. racemosa* and *E. caffra*, all extracts had LC_50_ values greater than 30 μg/mL.

**Table 4:** Cytotoxicity of acetone leaf extracts from nine species of the Fabaceae family on Vero monkey kidney cells and their selectivity index (SI) against bacterial strains

**Plant name**	**IC**_**50 **_**(μg/ml)**	**Selectivity index (LC**_**50 **_**/MIC)**					
		***SA***	***EF***	***EC***	***PA***	***BC***	***ST***
*Podalyria calyptrata*	131.98 ± 24.87^a^	<0.1	0.21	0.43	<0.3	<0.1	0.82
*Xylia torreana*	82.51 ± 9.46^b^	0.27	0.52	0.27	0.27	0.52	0.52
*Lonchocarpus nelsii*	81.09 ± 7.18^b^	<0.1	**1.01**	0.13	<0.1	0.13	**1.01**
*Indigofera cylindrica*	77.59 ± 6.10^b^	0.13	0.25	0.25	0.25	0.48	**1,94**
*Dalbergia nitidula*	51.28 ± 11.47^c^	0.32	0.32	0.32	0.17	0.64	0.32
*Crotalaria capensis*	45.47 ± 10.48^c^	0.07	0.57	0.07	0.07	0.15	**2.27**
*Virgilia divaricata*	30.08 ± 4.96^c^	0.1	0.05	0.1	<0.1	0.38	0.38
*Erythrina caffra*	19.93 ± 2.20^d^	<0.1	0.25	0.12	0.12	0.06	0.06
*Baphia racemosa*	10.7 ± 3.47^e^	0.03	0.07	0.02	<0.1	0.02	0.02
Doxorubicin	2.29 ± 1.04^f^	nd	nd	nd	nd	nd	nd

For IC_50_, n = 3, means values with different superscript letters are significantly different at p < 0.05; nd: not determined. In bold are interesting results with SI value >1. *SA: Staphylococcus aureus, EF: Enterococcus faecalis, BC*: *Bacillus cereus,* EC: *Escherichia coli*, *PA*: *Pseudomonas aeruginosa, ST*: *Salmonella typhimurium.*

In addition to ascertaining the likely safety of plant extracts for their potential use, standard cell-based toxicity assays are also performed *in vitro* at an early stage of the drug development process in order to remove high-risk materials [28]. Moreover, this helps to ensure that the biological activity of the plant extract is not due to a general metabolic toxic effect. Extracts of *C. capensis* and *L. nelsii* had SI values of 2.27 and 1.01 respectively. The higher the selectivity index is for a crude extract the more likely it is that the activity is not due to a general metabolic toxin [29]. Although elimination of toxic components by manipulation of the extract may yield more suitable antibacterial extracts [30] selection of an extract with a high selectivity index increases the potential that a useful herbal medicine can be produced.

## Conclusion

Acetone leaf extracts of *D. nitidula, X. torreana, C. capensis* and *I. cylindrica* had a relatively low cytotoxicity and a high antibacterial and/or antioxidant activity. Thus, these plant species could be considered as potential candidates for the development of novel antimicrobial formulations with reduced toxicity that can be used to promote animal health and productivity [31], as well as for further studies to isolate the active compounds [32]. These aspects are being followed up with some plant species in the Phytomedicine Programme.

## Competing interests

The authors declare that they have no competing interests.

## Authors’ contributions

JPD performed experiments and wrote the first draft of manuscript. LJM supervised the work and revised the manuscript; JNE initiated the project and revised the final manuscript. All authors read and approved the final manuscript.

## Pre-publication history

The pre-publication history for this paper can be accessed here:

http://www.biomedcentral.com/1472-6882/14/147/prepub
